# Identification of the Pyroptosis-Related Gene Signature for Overall Survival Prediction in Patients With Hepatocellular Carcinoma

**DOI:** 10.3389/fcell.2021.742994

**Published:** 2021-11-08

**Authors:** Shuang Liu, Ruonan Shao, Xiaoyun Bu, Yujie Xu, Ming Shi

**Affiliations:** ^1^Department of Hepatobiliary Oncology, Sun Yat-sen University Cancer Center, Guangzhou, China; ^2^State Key Laboratory of Oncology in South China, Collaborative Innovation Center for Cancer Medicine, Guangzhou, China

**Keywords:** pyroptosis, prognosis prediction, overall survival, hepatocellular carcinoma, immune infiltration

## Abstract

Hepatocellular carcinoma (HCC) is the second most lethal malignant tumor worldwide, with an increasing incidence and mortality. Due to general resistance to antitumor drugs, only limited therapies are currently available for advanced HCC patients, leading to a poor prognosis with a 5-year survival rate less than 20%. Pyroptosis is a type of inflammation-related programmed cell death and may become a new potential target for cancer therapy. However, the function and prognostic value of pyroptosis-related genes (PRGs) in HCC remain unknown. Here, we identified a total of 58 PRGs reported before and conducted a six-PRG signature *via* the LASSO regression method in the GEO training cohort, and model efficacy was further validated in an external dataset. The HCC patients can be classified into two subgroups based on the median risk score. High-risk patients have significantly shorter overall survival (OS) than low-risk patients in both training and validation cohorts. Multivariable analysis indicated that the risk score was an independent prognostic factor for OS of HCC patients. Functional enrichment analysis and immune infiltration evaluation suggested that immune status was more activated in the low-risk group. In summary, PRGs can be a prediction factor for prognosis of HCC patients and targeting pyroptosis is a potential therapeutic alternative in HCC.

## Introduction

Hepatocellular carcinoma (HCC) is the second most lethal malignant tumor worldwide with an increasing incidence (Torre et al., 2015). Due to lack of specific early symptoms and effective screening methods, a large portion of HCC patients have already reached advanced stage when they first diagnosed and lost the opportunity of surgery. Because of recurrent tendency and general resistance to chemotherapy, the prognosis of HCC patients remains poor (Bruix et al., 2014). Given that treatment failure of refractory HCC is largely due to resistance to drug-induced apoptosis, a further exploration of a new programmed cell death form is urgently needed to overcome drug resistance and provide more efficient prediction of overall survival (OS) in HCC.

Pyroptosis is a new identified form of cell death usually triggered by inflammasomes (Kesavardhana et al., 2020). Pyroptosis has particular morphological features to be distinguished from other types of cell death (Bergsbaken et al., 2009). Although DNA fragmentation and chromatin condensation are present during cell pyroptosis, their nucleus remains intact. Besides, pyroptotic cells undergo pore formation, osmotic lysis, and inflammatory factor release during the process (Shi et al., 2017). There are generally two modes of pytoptosis: canonical pathways triggered by caspase-1 and non-canonical pathways triggered by caspase 11 (murine) and caspase 4/5 (human beings) (Khanova et al., 2018). In the canonical pathway, inflammasomes recruit apoptosis-associated speck-like protein (ASC), which can activate caspase-1, resulting in cytokine secretion and gasdermin D (GSDMD) cleavage (Liu et al., 2016). The N-terminal of GSDMD forms pores in the plasma membrane, leading to the dysregulated discharge of inflammatory factors and cell lysis. As for the non-canonical inflammasome pathways, caspase-4/5 is activated by lipopolysaccharide to initiate cleavage of GSDMD and cell pyroptosis (Kovacs and Miao, 2017). Recently, a new pathway has been revealed, showing that some stimulations can activate caspase-3, inducing the cleavage of gasdermin E (GSDME) and the N-terminal of GSDME can also lead to the pore formation (Wang et al., 2017).

Accumulated evidence indicated that pyroptosis plays a key role in tumor progression and correlated with proliferation, migration, cell cycle, and drug resistance in multiple types of cancers (Heo et al., 2019; Yu et al., 2019; Tan et al., 2021). Distinct from apoptosis, pyroptotic cells release a large number of inflammatory cytokines and trigger a strong immune response, which may remodel the tumor-immune microenvironment (Frank and Vince, 2019). Carola et al. confirmed that sorafenib can induce pyroptosis in macrophages and unleash the NK-cell response in HCC (Hage et al., 2019). So, it may be a promising immunotherapy combination partner (Wu et al., 2021).

Considering that pyroptosis participates in the pathogenesis of tumor and may serve as a potential alternative in cancer therapy, a greater understanding of the underlying regulators of pyroptosis is therefore critical. Here we conducted this research to develop a pyroptosis-related gene signature to identify the expression level of these pyroptosis-related genes (PRGs) in normal liver and HCC tissues, improve the prognosis prediction of HCC, and explore the association between pyroptosis and immune microenvironment.

## Materials and Methods

### Sources of Hepatocellular Carcinoma Datasets

The workflow chart (Supplementary Figure 1) shows samples used at each stage of analysis. The microarray data of two HCC cohorts, GSE14520 and GSE76427, were obtained from a publicly dataset in the GEO database.^1^ Samples without survival data were excluded from further analysis. The relative expression of genes was adjusted with the ‘‘limma’’ R package. In addition, pyroptosis-related genes were obtained in the REACTOME_PYROPTOSIS and GOBP_PYROPTOSIS gene set from the MSigDB database^2^ and prior reviews (Man and Kanneganti, 2015; Wang and Yin, 2017; Karki and Kanneganti, 2019; Ye et al., 2021). All datasets used in this study are publicly available.

### Identification of Differentially Expressed Pyroptosis-Related Genes

The “limma” package was utilized to screen differently expressed genes (DEGs) (*p* value < 0.05) between HCC and normal liver. Gene cloud biotechnology information (GCBI) was employed to assess relationships between model-linked and other related proteins. The genetic alternations of the six genes were obtained from the cBioPortal website.^3^

### Construction and Verification of the Pyroptosis-Related Genes Prognostic Model

The GSE14520 cohort was selected as the training cohort for the generation of the prognostic model. PRGs significantly associated with HCC prognosis were identified (*p* value < 0.05) *via* univariate Cox analysis. LASSO is a popular machine learning algorithm, which was extensively utilized in medical studies (Liu et al.2021a-d). Here, the LASSO regression was used to develop the prognostic signature. The prognostic model was based on 10-fold cross-validation estimator penalty maximum likelihood estimation. Moreover, the minimum criteria of the penalized maximum likelihood estimator were used to identify the quintessential penalty parameter λ values. The HCC patients were assigned into either a low- or high-risk subgroup based on the median risk score for further analysis. The predictive efficacy of the PRG model was tested by a time-dependent ROC curve. The GSE76427 cohort was selected as the validation cohort. The risk scores of participants were computed with the unified formula established in the training cohort.

### Functional Enrichment Analysis

GSEA v4.0.2 software (see text footnote 2) and c2.cp.kegg.v7.0.symbols gene sets are utilized to explore possible biological functions related to risk scores. Normalized *p*-value < 0.05 was considered significant. Moreover, we computed the enrichment score of 16 kinds of immune cells and 13 immune-linked networks through ssGSEA.

### Statistical Analysis

Categorical data were analyzed by the Pearson chi-square test. Survival analysis between two subgroups was performed *via* Kaplan–Meier analysis and the log-rank test. Univariate and multivariate Cox analyses were utilized to identify independent prognostic factors for HCC. The ROC curve was used to evaluate the predictive power of the PRG profile, followed by calculation of the AUC. The confidence interval (CI) was calculated *via* the bootstrap formula. R software (Version 3.6.0) and SPSS (Version 24.0) was used for all data analyses. A two-sided *p* < 0.05 was set as the significance threshold.

## Results

### Identification of Prognostic Pyroptosis-Related Genes Between Hepatocellular Carcinoma and Normal Liver Tissues

**FIGURE 1:**
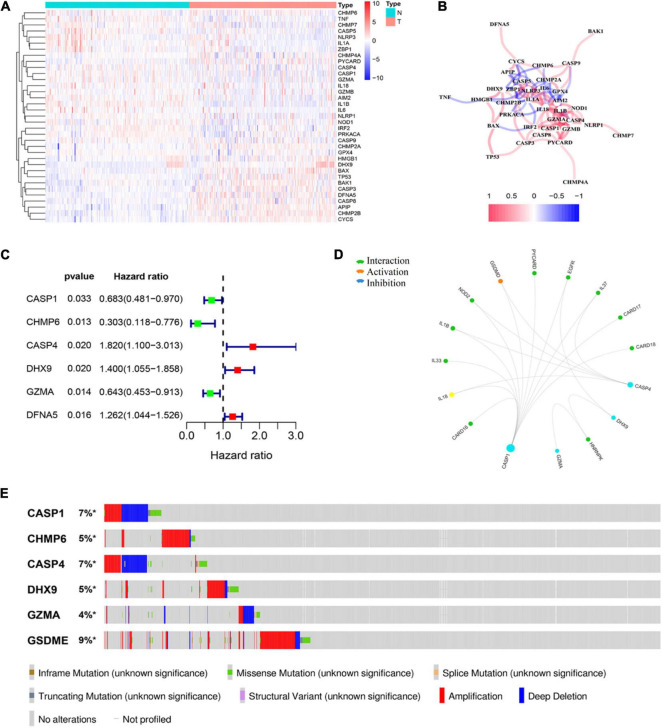
Identification of prognostic differently expressed genes (DEGs) between hepatocellular carcinoma (HCC) and normal liver tissues. **(A)** Heat map of differently expressed pyroptosis-related genes (PRGs) between the normal liver and HCC tissues. **(B)** The correlation network of candidate PRGs. **(C)** Forest plots showing the results of the univariate Cox analysis between gene expression and overall survival (OS). **(D)** Protein–protein interaction (PPI) analysis to explore the potential association among the prognostic DEGs. **(E)** The landscape of genetic alteration profiles of six genes in CCLE, obtained from the cBioPortal website.

A total of 58 pyroptosis-related gene expression levels were evaluated in 221 HCC and 220 normal liver tissues in the GSE14520 cohort (Supplementary Table 1). Among them, we identified 35 DEGs, including 13 upregulated genes (CHMP2A, GPX4, HMGB1, DHX9, BAX, TP53, BAK1, CASP3, DFNA5, CASP8, APIP, CHMP2B, and CYCS) and 22 downregulated genes (CHMP6, TNF, CHMP7, CASP5, NLRP3, IL1A, ZBP1, CHMP4A, PYCARD, CASP4, CASP1, GZMA, IL18, GZMB, AIM2, IL1B, IL6, NLRP1, NOD1, IRF2, PRKACA, and CASP9) in HCC tissue. The result of the differential expression analysis is shown as a heat map (Figure 1A). The correlation network of DEGs is shown in Figure 1B. Univariate Cox regression analysis was utilized to screen prognosis-related genes. The six DEGs (CASP1, CHMP6, CASP4, DHX9, GZMA, and DFNA5) were identified to be significantly associated with OS (Supplementary Figure 2). Among them, three genes (CASP4, DHX9, and DFNA5) were classified as risk factors, while the other three genes (CASP1, CHMP6, and GZMA) were defined as protective factors (Figure 1C). Then, we explore the potential association among the prognostic PRGs through GCBI analysis (Figure 1D). The mutation profile of six DEGs in tumor cells is shown in Figure 1E.

### Establishment of a Prognostic Pyroptosis-Related Genes Model in the GEO Cohort

**FIGURE 2:**
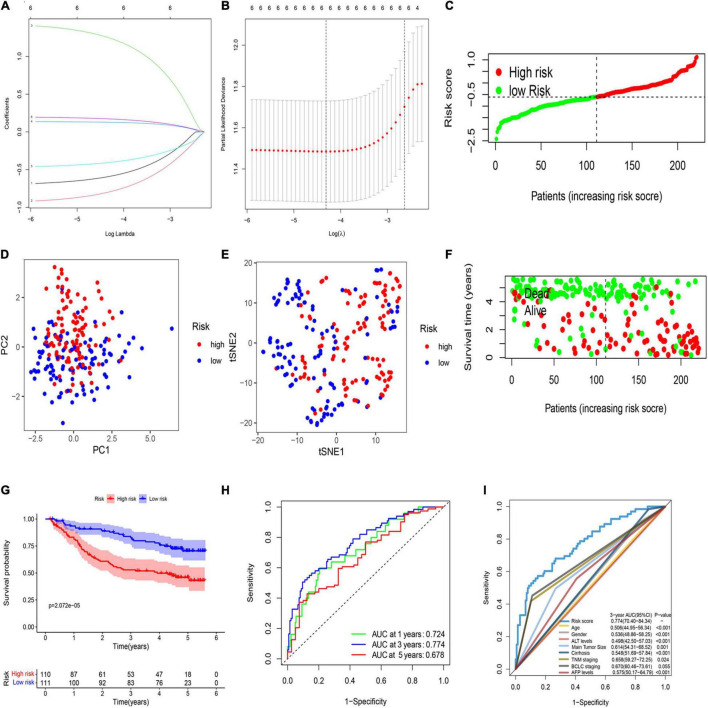
Construction of a prognostic PRG model in the GEO cohort. **(A)** Validation for LASSO regression *via* minimum criteria. **(B)** LASSO coefficients of pyroptosis-related genes. **(C)** Risk score analysis of the six-PRG profile in the training cohort. **(D)** PCA plot based on the risk score. **(E)** t-SNE analysis of the training cohort. **(F)** The survival status analysis based on risk score in the training cohort. **(G)** The Kaplan–Meier curve of the low- and high-risk groups based on the six-pyroptosis-related gene profile. **(H)** Time-dependent ROC analysis for the 1-, 3-, and 5-year OS of the six-PRG profile in the training cohort. **(I)** Time-dependent receiver operating characteristic (ROC) analysis for 3-year OS of six-pyroptosis-related genes was compared to age, gender, ALT levels, main tumor size, cirrhosis, TNM stage, BCLC stage, and AFP levels in the training cohort.

LASSO regression analysis was used to establish the best-weighting coefficients of the six prognostic PRGs. The signature was identified according to the minimum criterion optimal λ value (Figures 2A,B). The formula used for risk score computation was as follows: risk score = (–0.591 × CASP1) + (–0.795 × CHMP6) + (1.246 × CASP4) + (0.128 × DHX9) + (–0.405 × GZMA) + (0.172 × DFNA5). A total of 221 patients were separated into either low- or high-risk subgroup based on the median threshold calculated by the risk score model (Figure 2C). Principal component analysis (PCA) and t-distributed stochastic neighbor embedding (t-SNE) analysis indicated that patients in the two groups were well separated (Figures 2D,E). Patients with a low risk score had less deaths and longer OS than those with high risk (Figure 2F). The Kaplan–Meier analysis showed that patients in the low-risk group exhibited a significantly better OS than high-risk patients (Figure 2G). Then, we evaluated the efficacy of the prognostic model through time-dependent receiver operating characteristic (ROC) analysis. The area under the ROC curve (AUC) for 1-, 3-, and 5- year OS was 0.724, 0.774, and 0.678, respectively (Figure 2H). We also compared several other clinical prognostic models to evaluate the efficacy of the PRG model. The analyses showed that our model was better than other models for prognosis prediction (Figure 2I and Supplementary Figure 3).

### Validation of the Prognostic Pyroptosis-Related Gene Signature in the External Dataset

**FIGURE 3:**
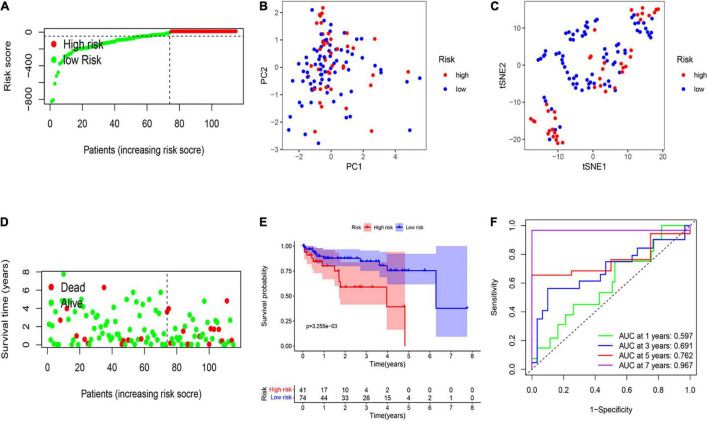
External validation of the risk signature. **(A)** Risk score analysis of the six-PRG profile in the validation cohort. **(B)** PCA plot based on the risk score. **(C)** t-SNE analysis of the validation cohort. **(D)** The survival status analysis for each patient based on the risk score in the validation cohort. **(E)** Kaplan–Meier curve of low- and high-risk groups based on the six-pyroptosis-related gene profile. **(F)** Time-dependent ROC analysis for the 1-, 3-, 5-, and 7-year OS of the six-pyroptosis-related gene profile in the validation cohort.

A total of 115 HCC patients from the GEO cohort (GSE76427) were applied as a validation dataset. Based on the threshold in the training cohort, 115 patients were assigned into the low-risk (*n* = 74) and high-risk groups (*n* = 41; Figure 3A). The result of PCA and t-SNE analysis was similar to the training cohort (Figures 3B,C). Patients with high risk were proved to have shorter OS and higher death rates than those with a low risk score (Figure 3D). The Kaplan–Meier curve also revealed a significant difference in the OS between two subgroups (Figure 3E). The AUCs were respectively 0.597, 0.691, 0.762, and 0.967 in the 1-, 3-, 5-, and 7-year OS in the validation set, indicating that the model had a good predictive efficacy (Figure 3F).

### Identification of the Pyroptosis-Related Genes Risk Model as an Independent Prognostic Predicting Factor for Overall Survival

**FIGURE 4:**
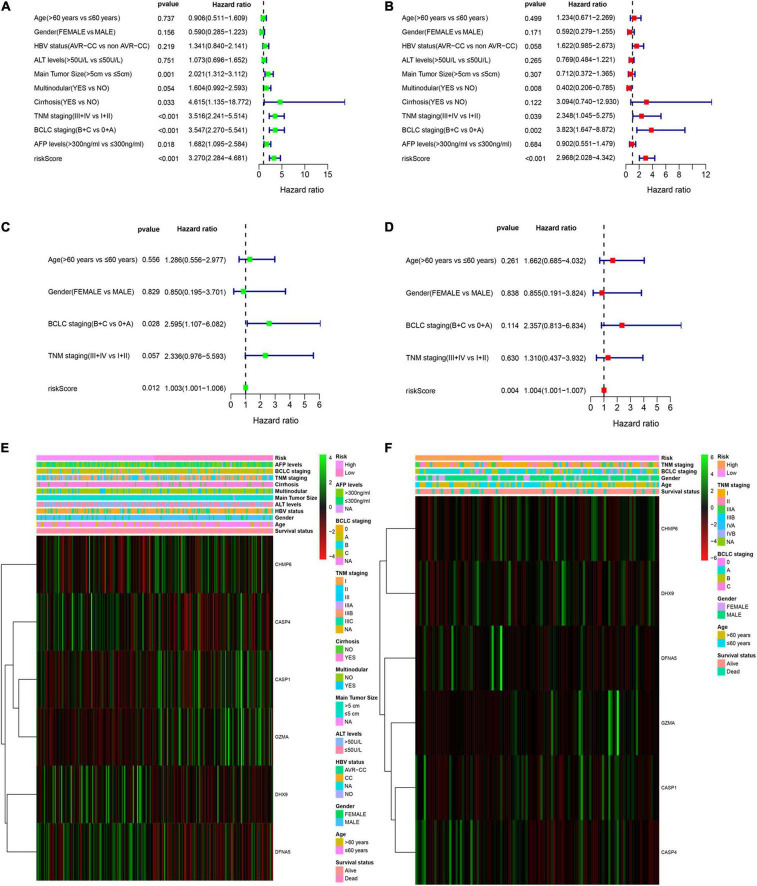
Independent prognostic value of the PRG risk model. **(A,B)** Univariate **(A)** and multivariate Cox **(B)** analyses in the training cohort. **(C,D)** Univariate **(C)** and multivariate Cox **(D)** analyses in the validation cohort. **(E,F)** Heatmap of the six-pyroptosis-related gene profile and clinical features in two subgroups for the training **(E)** and validation **(F)** cohorts.

Multivariable Cox regression analyses for risk score and other confounding factors were performed in two cohorts. As shown in Results, the univariate Cox regression analyses indicated that the risk model was an independent prognostic indicator for OS in both training and validation cohorts (Figures 4A,C). After considering other clinical factors, the risk score was still a prognostic factor through multivariable Cox regression analyses in both two cohorts (Figures 4B,D). Besides, we explored clinical features of two subgroups and found that clinical characteristics are different between the low- and high-risk groups (Supplementary Table 2). We noticed that patients with early HCC were more prominent in the low-risk group, while advanced patients were more easily to be classified into the high-risk group. This may explain why the low-risk group tended to show a better survival status. Both two cohorts showed similar results (Figures 4E,F).

### Validation of the Nomogram for Overall Survival in the Training Cohort

**FIGURE 5:**
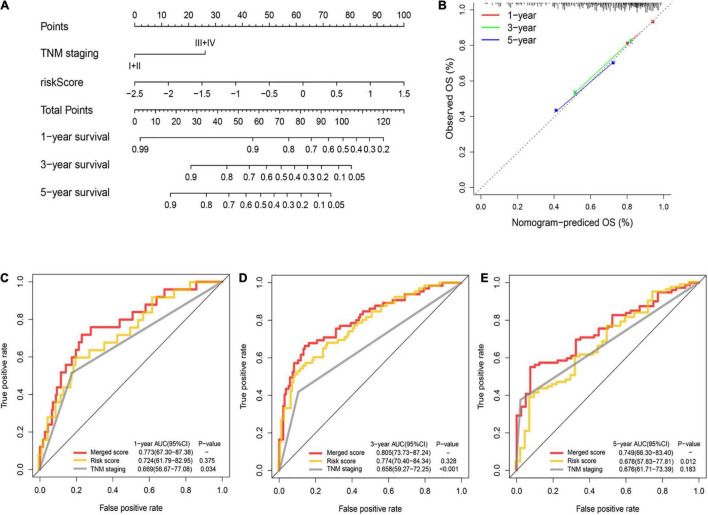
Construction and validation of the nomogram to predict the overall survival in the training cohort. **(A)** Nomogram built based on TNM stage and risk score in the training cohort. **(B)** Calibration plot of the nomogram. **(C–E)** Time-dependent ROC curves of nomograms were compared based on 1- **(C)**, 3- **(D)**, and 5-year **(E)** OS in the training cohort.

A nomogram was utilized for integration of the TNM stage and PRG profile in the training cohort (Figure 5A). The calibration plots suggested that the 1-, 3-, and 5-year OS can be estimated with great precision (Figure 5B). The AUC of the merged scores was proved higher than the TNM stage, suggesting that the nomogram can enhance the OS prediction compared with standard prognostic factors (Figures 5C–E).

### Signal Pathway Enrichment Analysis Based on the Risk Model

**FIGURE 6:**
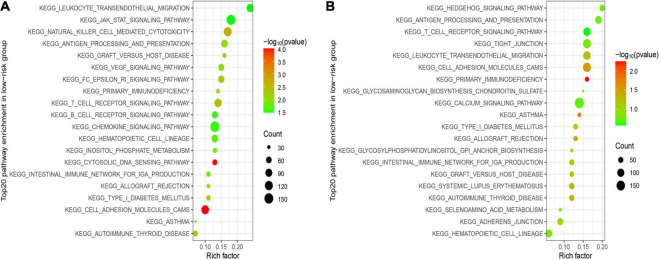
Functional enrichment analysis between two subgroups. **(A)** Top 20 significantly enriched KEGG pathways in the LR group of the training cohort. **(B)** Top 20 significantly enriched KEGG pathways in the LR group of the validation cohort.

GSEA was utilized in two cohorts to distinguish the biological functions and networks related to risk score. The results indicated that a major of DEGs were involved in immune-related and chemokine-mediated signaling pathways (Figures 6A,B). The NK-cell-mediated cytotoxicity, antigen processing and presentation, and T cell and B cell receptor signaling pathway were more activated in the low-risk subgroup.

### The Relationship of Risk Score and Immune Status

**FIGURE 7:**
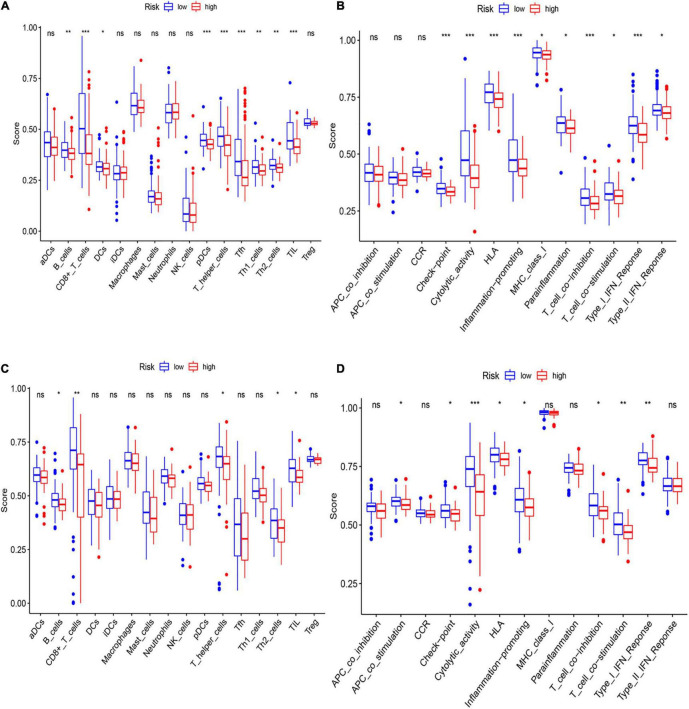
Analysis of immune status between low- and high-risk subgroups. **(A,B)** The enrichment scores of 16 immune cells **(A)** and 13 immune-related functions **(B)** of two subgroups in the training cohort. **(C,D)** The enrichment scores of 16 immune cells **(C)** and 13 immune-related networks **(D)** of two subgroups in the validation cohort.

To further asses the immune status in the two subgroups, 16 kinds of immune cells with 13 immune-linked functions were examined through employing the single-sample gene set enrichment analysis (ssGSEA). We found that the scores of CD8+ T cells, pDCs, Th cells, and TILs in the low-risk subgroup were remarkably higher than in the high-risk group in the training set (Figure 7A). Moreover, except for the APC and CCR pathways, the other 10 immune pathways all represented higher activity in the low-risk subgroup (Figure 7B). The exploration of the validation cohort also showed similar results (Figures 7C,D). In conclusion, our result suggested that HCC patients in the low-risk subgroup have more activated immune status than the high-risk subgroup.

## Discussion

Pyroptosis is a novel mechanism of programmed cell death accompanied by release of inflammatory factors and immune responses. It has been reported that the effects of pyroptosis seems different in multiple cancers (Xia et al., 2019). On the one hand, pyroptosis induced by antitumor drugs can inhibit the development of cancer and thereby can serve as a potential target in tumor therapy; on the other hand, pyroptotic cells release plenty of inflammatory factors, resulting in drug resistance to chemotherapy agents and malignant transformation of normal cells. Pyroptosis also functions in HCC progression. Wang et al. confirmed that pyroptosis-related regulator NLRP3 was remarkably downregulated in HCC compared with normal liver and negatively correlated with pathological grades. Besides, 17β-estradiol exerted antitumor effects through activating NLRP3 inflammasome and triggering pyroptosis (Wei et al., 2019). The AIM2 inflammasome can inhibit HCC tumor growth by targeting the mTOR signal and inducing pyroptosis (Lozano-Ruiz and Gonzalez-Navajas, 2020). Drug resistance has been a substantial clinical challenge for so long. Apoptosis-resistant tumor cells are usually less sensitive to chemotherapy agents. New forms of cell-programmed death induced by drugs would be of great value in cancer treatment. Evidence indicated that some antitumor drugs or molecules could trigger pyroptosis in HCC. It has been reported that miltirone reduced intracellular reactive oxygen species (ROS), leading to cell pyroptosis, and inhibited tumor growth through suppressing phosphorylation of MEK and ERK1/2 in the HCC (Zhang et al., 2020). In a word, pyroptosis is a promising therapeutic target for HCC management. A prognostic PRG signature has already been constructed in ovarian cancer (Ye et al., 2021). However, the prognostic value and the role of PRG in HCC have not been defined yet. We then performed this research to demonstrate the signature of PRG and their potential functions in HCC.

Here in this research, we first evaluate the expression level of 58 previously reported PRGs. We found that 13 PRGs (CHMP2A, GPX4, HMGB1, DHX9, BAX, TP53, BAK1, CASP3, DFNA5, CASP8, APIP, CHMP2B, and CYCS) were upregulated in HCC, while 22 genes (CHMP6, TNF, CHMP7, CASP5, NLRP3, IL1A, ZBP1, CHMP4A, PYCARD, CASP4, CASP1, GZMA, IL18, GZMB, AIM2, IL1B, IL6, NLRP1, NOD1, IRF2, PRKACA, and CASP9) were downregulated compared with normal liver.

Then we generated a risk signature prognostic model *via* univariate Cox analysis and LASSO regression analysis, in which six PRGs (CASP 1, CHMP6, DHX9, GAZMA, DFNA5, and CASP4) were included. The efficiency of the model was well validated through external datasets. CASP1 is the key regulator of canonical inflammasome pathways in pyroptosis. It can cleave GSDMD protein into two parts, and the N-terminal fragment of GSDMD forms the pole on the membrane, leading to cell swelling and lysis. Caspase 1 is downregulated in HCC tissues compared with normal liver, indicating that the canonical pyroptosis pathway is possibly inhibited in the pathogenesis of HCC (Zhang et al., 2019). Our results also confirm that CASP 1 is a protective factor and prognostic biomarker of HCC. Drug-induced caspase 1 activation may be an alternative treatment for HCC. However, several reports also pointed out that the proinflammatory effect of caspase 1 may on the other side facilitate tumor progression. The transform value of targeting caspase 1 in cancer treatment still needs further exploration. CHMP6 is an essential component of the endosomal sorting required for transport complex III (ESCRT-III) which is involved in the degradation of membrane proteins. Wang et al. found that overexpression of CHMP6 induced cell apoptosis in cervical carcinoma (Fu et al., 2009). However, the role of CHMP6 in HCC remains unknown. We revealed that the expression level of CHMP6 was associated with favorable prognosis, which may be due to its regulation of pyroptosis. DHX9 is an NTP-dependent helicase protein function to unwind both RNA and DNA. It plays a central role in many important biological processes, including gene transcription and translation, RNA processing and transport, the regulation of DNA replication, and genome stability maintenance (Wang et al., 2019). DHX9 is overexpressed in various types of cancers, including HCC, which is confirmed in our study. The pro-oncogenic role of DHX9 may partly be due to its regulation of PRGs. GZMA is traditionally defined to be death inducers in target cells. Besides, GZMA could also regulate inflammation response and cytokine production. Nakamura et al. reported that melanoma harboring high GZMA may respond preferentially to nivolumab treatment, which may be because GZMA could enhance pyroptosis of tumor cell (Inoue et al., 2016). DFNA5 encodes GSDME protein cleaved by caspase 3 and directly induces pyroptosis through punching holes in the membrane (Rogers et al., 2017). Our results showed that DFNA5 was increased in HCC tissue and correlated with poor outcomes, indicating that it functioned as a tumor-promoting factor. Further exploration is needed to find out whether/how DFNA5 exerts its effect in pyroptosis and tumorigenesis. Caspase 4 is involved in innate immunity as well as in promoting maturation and secretion of inflammatory cytokines (Shi et al., 2014). Caspase 4 is the major mediator of non-canonical pathways of pyroptosis. Interestingly, evidence indicated that the CASP4 gene participated in cell migration and invasion in epithelial cancer. Our results also suggested that CASP4 was upregulated in HCC and seemed to act as a cancer-promoting gene. More evidence is required to reveal the function of CASP4 in pyroptosis and tumor promotion. In summary, three genes in the PRG model (CASP1, CHMP6, and GZMA) is shown to be a protective factor, while the other three genes (CASP4, DHX9, and DFNA5) were defined as tumor-promoting factors.

To further explore the biological functions associated with risk score, we revealed that DEGs between two risk subgroups were mainly involved in immune responses and chemokine-mediated signaling pathways, indicating that pyroptosis regulated the tumor immune microenvironment through inflammatory responses. The antitumor immune cells and pathways were mostly downregulated in the high-risk group, suggesting an impairment immune function and leading to poor prognosis. In conclusion, these findings showed that poor outcome of high-risk patients may partly be due to the inhibited state of antitumor immunity.

In summary, we performed a systematic bioinformatics analysis to identify the signature of six pyroptosis-related prognostic genes (CASP1, CHMP6, GZMA, CASP4, DHX9, and DFNA5) in HCC cohorts. Our model has independent prognostic value of OS for HCC patients. We demonstrated that pyroptosis may affect the biological behaviors of tumor through regulating immune activity.

## Data Availability Statement

The original contributions presented in the study are included in the article/Supplementary Material, further inquiries can be directed to the corresponding author.

## Ethics Statement

Ethical review and approval was not required for the study on human participants in accordance with the local legislation and institutional requirements. Written informed consent for participation was not required for this study in accordance with the national legislation and the institutional requirements.

## Author Contributions

SL and RS designed the research. RS and XB performed the data analysis. SL and YX wrote the manuscript. MS reviewed the manuscript and supervised the study. All authors read and approved the final manuscript.

## Conflict of Interest

The authors declare that the research was conducted in the absence of any commercial or financial relationships that could be construed as a potential conflict of interest.

## Publisher’s Note

All claims expressed in this article are solely those of the authors and do not necessarily represent those of their affiliated organizations, or those of the publisher, the editors and the reviewers. Any product that may be evaluated in this article, or claim that may be made by its manufacturer, is not guaranteed or endorsed by the publisher.

## Footnotes

1
http://www.ncbi.nlm.nih.gov/geo/

2
http://software.broadinstitute.org/gsea/login.jsp

3
http://www.cbioportal.org/
