# Supplementary material for: Identification of the Pyroptosis-Related Gene Signature for Overall Survival Prediction in Patients With Hepatocellular Carcinoma
Source: Front Cell Dev Biol. 2021 Nov 8;9:742994. doi: 10.3389/fcell.2021.742994 (PMC8606528; doi:10.3389/fcell.2021.742994)
Supplement: Supplementary file 1 [file Data_Sheet_1.PDF]

Supplementary Material

1 Supplementary Figures and Tables

Supplementary figure1: Flow chart of data collection and analysis.

Supplementary figure2: Survival analyses of each DE PRG in the training cohort.

Supplementary figure3: 1- and 5-year AUC of risk score and other clinical prognostic factors.

Supplementary table1: 58 pyroptosis-related genes.

Supplementary table2: Differently expressed PRGs between HCC and normal liver.

1.1 Supplementary Figures

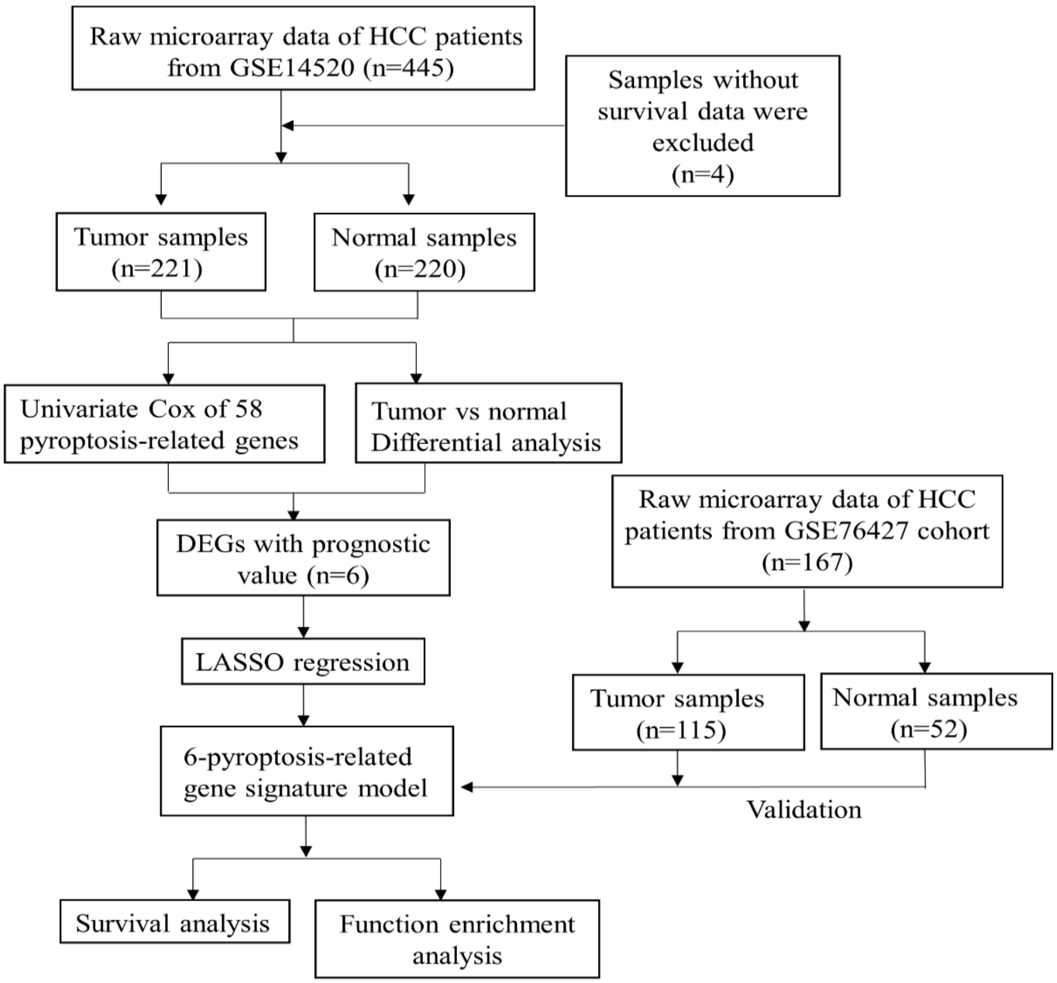

Supplementary Figure 1. Flow chart of data collection and analysis.

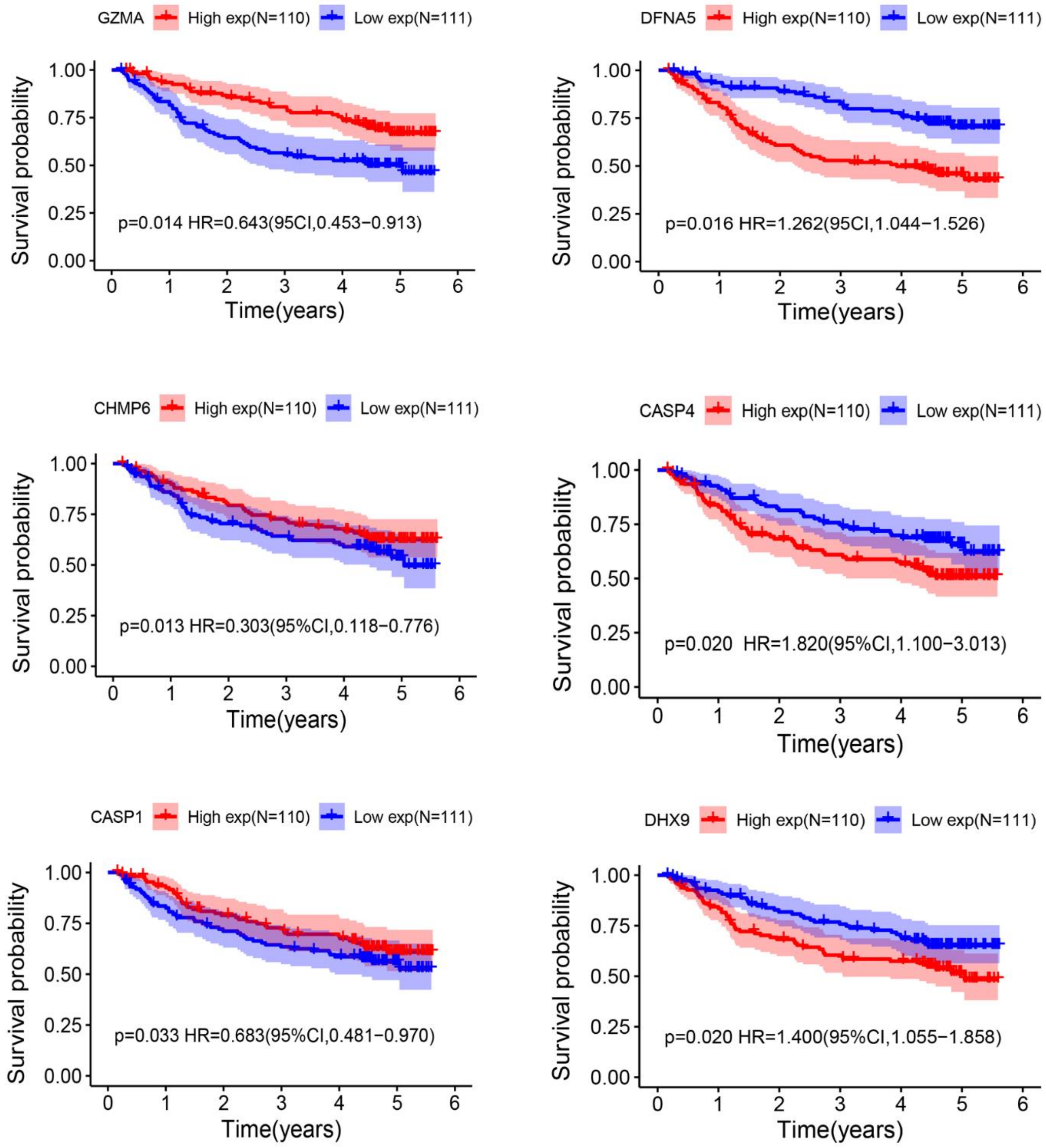

Supplementary Figure 2. Survival analyses of each DE PRG in the training cohort.

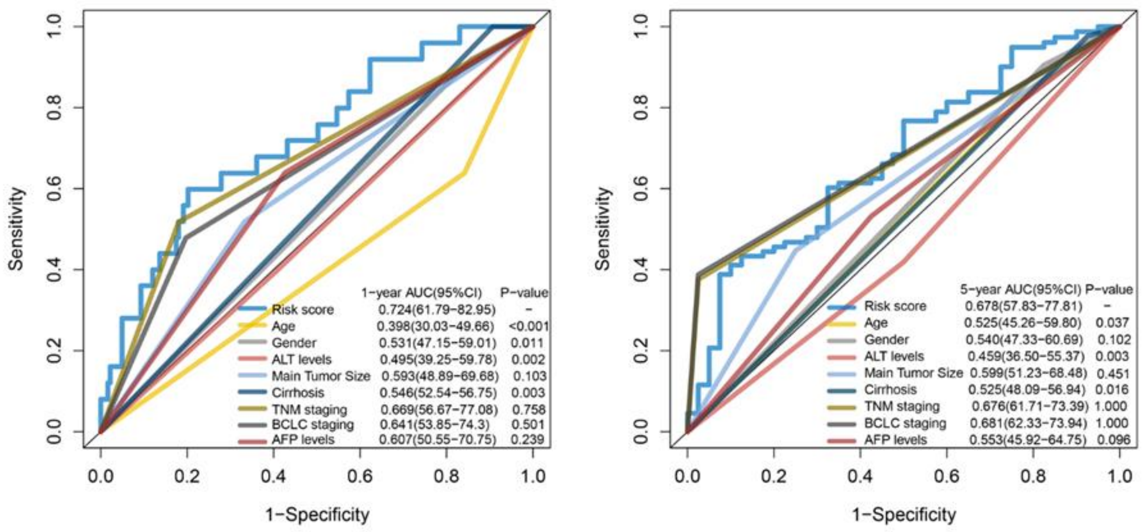

Supplementary Figure 3. 1- and 5-year AUC of risk score and other clinical prognostic factors.

Supplementary table1. 58 pyroptosis-related genes.

Genes	Full-names
AIM2	Absent in melanoma 2
CASP1	cysteine-aspartic acid protease-1
CASP3	cysteine-aspartic acid protease-3
CASP4	cysteine-aspartic acid protease-4
CASP5	cysteine-aspartic acid protease-5
CASP6	cysteine-aspartic acid protease-6
CASP8	cysteine-aspartic acid protease-8
CASP9	cysteine-aspartic acid protease-9
ELANE	elastase, neutrophil expressed
GPX4	glutathione peroxidase 4
GSDMA	gasdermin A
GSDMB	gasdermin B
GSDMC	gasdermin C
GSDMD	gasdermin D
GSDME	gasdermin E
IL18	interleukin 18
IL1B	interleukin 1 beta
IL6	interleukin 6
NLRC4	NLR family CARD domain containing 4
NLRP1	NLR family pyrin domain containing 1
NLRP2	NLR family pyrin domain containing 2
NLRP3	NLR family pyrin domain containing 3
NLRP6	NLR family pyrin domain containing 6
NLRP7	NLR family pyrin domain containing 7
NOD1	nucleotide binding oligomerization domain containing 1
NOD2	nucleotide binding oligomerization domain containing 2
PJVK	pejvakin/deafness, autosomal recessive 59
PLCG1	phospholipase C gamma 1
PRKACA	protein kinase cAMP-activated catalytic subunit alpha
PYCARD	PYD and CARD domain containing
SCAF11	SR-related CTD associated factor 11
TIRAP	TIR domain containing adaptor protein
TNF	tumor necrosis factor
BAK1	BCL2 antagonist/killer 1
TP63	tumor protein p63
CHMP2B	charged multivesicular body protein 2B
BAX	BCL2 associated X, apoptosis regulator
GZMB	granzyme B
CHMP4B	charged multivesicular body protein 4B
IL1A	interleukin 1 alpha
CHMP3	charged multivesicular body protein 3
IRF1	interferon regulatory factor 1

CHMP2A	charged multivesicular body protein 2A
TP53	tumor protein p53
CHMP7	charged multivesicular body protein 7
CHMP4C	charged multivesicular body protein 4C
IRF2	interferon regulatory factor 2
CYCS	cytochrome c, somatic
CHMP6	charged multivesicular body protein 6
HMGB1	high mobility group box 1
CHMP4A	charged multivesicular body protein 4A
DHX9	DExH-box helicase 9
GZMA	granzyme A
NLRP9	NLR family pyrin domain containing 9
NAIP	NLR family apoptosis inhibitory protein
APIP	APAF1 interacting protein
ZBP1	Z-DNA binding protein 1
DFNA5	DFNA5, deafness associated tumor suppressor

Supplementary table2. Differently expressed PRGs between HCC and normal liver in training cohort.

gene	conMean	treatMean	logFC	pValue	FDR
BAK1	4.4481	4.674036	0.071479	1.01E-13	3.66E-13
CHMP2B	5.9189	6.523015	0.14021	3.22E-28	3.22E-27
BAX	3.988143	4.330798	0.118916	1.17E-12	3.36E-12
GZMB	3.993073	3.93772	-0.02014	2.00E-07	3.81E-07
IL1A	3.579575	3.475371	-0.04262	5.59E-10	1.32E-09
IL1B	4.284802	3.915649	-0.12998	6.20E-30	8.27E-29
CHMP2A	8.405764	8.803573	0.06671	2.11E-21	1.69E-20
CASP1	5.015265	4.838247	-0.05184	0.000135	0.000193
CASP5	3.598159	3.49984	-0.03997	1.75E-06	2.80E-06
TP53	4.31793	4.597291	0.090444	5.52E-08	1.10E-07
CHMP7	5.944995	5.823036	-0.0299	0.001113	0.001536
IL18	3.739177	3.645111	-0.03676	7.64E-07	1.27E-06
CASP3	5.216586	5.646924	0.114359	8.02E-20	5.35E-19
IRF2	5.844382	5.497124	-0.08837	7.21E-14	2.88E-13
CYCS	9.445764	10.13627	0.101788	2.19E-33	4.39E-32
CHMP6	4.203686	4.250956	0.016132	0.008224	0.009399
HMGB1	10.52505	10.59188	0.009131	0.035334	0.03926
CASP4	4.663432	4.557628	-0.03311	0.005729	0.00674
CHMP4A	6.520695	6.583171	0.013757	0.001461	0.001885
DHX9	4.801615	5.185579	0.110985	6.26E-19	3.58E-18
NLRP1	4.1747	4.077452	-0.034	3.58E-13	1.19E-12
GZMA	4.423436	4.197711	-0.07556	2.43E-07	4.43E-07
APIP	5.800609	6.599133	0.186072	7.75E-42	3.10E-40
ZBP1	3.773805	3.6372	-0.05319	3.52E-06	5.42E-06
CASP8	5.271261	5.521191	0.066831	9.23E-13	2.84E-12
AIM2	3.983877	3.806044	-0.06588	3.85E-10	9.62E-10
CASP9	5.296661	5.224333	-0.01984	0.002435	0.002952
GPX4	10.28033	10.10753	-0.02446	0.001317	0.001756
IL6	3.621605	3.4188	-0.08314	3.42E-09	7.21E-09
NLRP3	3.516627	3.439622	-0.03194	2.62E-07	4.56E-07
NOD1	4.668795	4.583498	-0.0266	0.00217	0.002713
PRKACA	6.296482	5.998711	-0.06989	2.25E-12	6.00E-12
PYCARD	5.255577	5.739191	0.126998	2.00E-05	2.96E-05
TNF	3.649786	3.532462	-0.04714	9.73E-10	2.16E-09
DFNA5	5.277127	5.996044	0.184258	1.75E-14	8.77E-14
